# The psychometric properties of a new outcome measure of resilience for people living with dementia: The Bangor Dementia Resilience Scale

**DOI:** 10.1186/s40359-025-02695-z

**Published:** 2025-04-15

**Authors:** Jennifer Rhiannon Roberts, Catherine Anne MacLeod, Gill Windle, Zoe Hoare, Joshua Stott, Mary Pat Sullivan, Paul M. Camic, Emilie V. Brotherhood, Sebastian J. Crutch, on behalf of the Rare Dementia Support (RDS) Research Team

**Affiliations:** 1 DSDC Wales Research Centre, School of Health Sciences, Bangor University, Ardudwy, Normal Site, Bangor, LL57 2PZ UK; 2 Centre for Population Health Sciences, Usher Institute, the University of Edinburgh, Edinburgh, EH16 4UX UK; 3 Department of Clinical, Educational and Health Psychology, University College London (UCL), London, WC1E 6BT UK; 4 Faculty of Education and Professional Studies, School of Social Work, Nipissing University, Nipissing, North Bay, ON Canada; 5 Dementia Research Centre, Queen Square Institute of Neurology, University College London (UCL), London, WC1N 3AR UK

**Keywords:** Dementia, Resilience, Outcome measure, Person-centred, Strengths-based, Measurement, Adaptation

## Abstract

**Background:**

Psychometrically sound resilience outcome measures are essential to establish how health and care services or interventions can enhance the resilience of people living with dementia. In response to a lack of resilience outcome measures designed specifically with, and for, people living with dementia, this research builds on several stages of measurement development and evaluates the psychometric properties of a new outcome measure of resilience for people living with dementia.

**Methods:**

We aimed to recruit 185 people aged 18 + living with dementia. An online survey containing demographic questions, the draft 37-item resilience measure, the 7-item Generalised Anxiety Disorder Assessment (GAD- 7) and the 5-item Canterbury Wellbeing Scale was widely shared in the UK. Three people living with dementia piloted the survey before recruitment commenced and gave suggestions for improvement. Exploratory factor analysis was applied to the draft resilience measure and the construct validity and internal consistency ascertained. Convergent validity with other measures was tested.

**Results:**

Minor changes were made to the survey following piloting to help people with dementia. The survey was completed by 193 participants, aged 47–93 (M = 69.9; *SD* = 9.5), 58% male, and living with a range of dementia diagnoses. The exploratory factor analysis led to a final 19-item measure (Chronbach’s Alpha = 0.85) with 5-factors underlying resilience: ‘Outlook’, ‘Adaptation’, ‘Acceptance’, ‘community and peer support’ and ‘family and friends’. The new resilience measure demonstrated convergent validity with well-being (r = 0.49, *p* < 0.001) and anxiety (r = - 0.28, *p* < 0.001).

**Conclusions:**

This study presents preliminary field-testing and validation of the Bangor Dementia Resilience Scale, a new psychometrically sound resilience measure for people living with mild to moderate dementia. The scale may be a valuable tool for practitioners to provide strengths-based and person-centred support to maintain and enhance the resilience of people living with dementia, and evaluating the extent to which health and social care services may improve resilience. Given the global policy focus to support people with dementia to live as well as possible, the new scale has international significance for translation and cultural adaptation by other countries.

## Introduction

Presently over 55 million people are living with dementia globally [1], and this figure is forecasted to surpass 150 million by 2050 [2]. As such, dementia is considered a major public health concern internationally [3]. In the current absence of a cure and with limited medical treatments available, enabling people to ‘live well’ with dementia is a priority [4]. Accordingly, internationally, policies promote well-being and independence by using strengths-based approaches to supporting people requiring care or support [5–8].

‘Living well’ with dementia has been associated with the concept of resilience [9], which puts a focus on an individual’s strengths rather than deficits. There is growing interest in the importance of resilience within international policy, and building resilience is a key priority of Health 2020, the European policy framework [10, 11]. This is moving on from the historical tendency to focus solely on deficits and disregarding the importance of also considering the strengths and assets available [12].

The ecological resilience framework and the WHO European policy framework for health and wellbeing suggest that resilience can be achieved by drawing on individual, community and societal resources [11, 13]. Resilience is regarded by the WHO as being ‘essential for modernizing and increasing the performance of health services and public health programmes’ [11: pp. 8]. In order to achieve these goals, it is essential to be able to measure resilience but, despite this, a systematic review and psychometric evaluation of resilience measurement scales found that no established resilience measure had been designed specifically with and for people living with dementia, which means it is not possible to evaluate the impact of services and interventions on their resilience [14]. The review concluded further work is required.

In response to this need, we are developing a psychometrically robust resilience measure for people living with dementia, that is appropriate for evaluating the impact of health, psychological and social care services and interventions. The work follows rigorous methodology for developing health measurement scales proposed by Streiner, Norman and Cairney [15].

First, we developed a conceptual model of resilience in people living with dementia by exploring the limited published research and speaking to people living with dementia and their carers [16]. This work established resilience in dementia as a multi-level construct (Table 1) which is not reflected in existing validated resilience measures [14]. Second, we generated a draft resilience outcome measure using the conceptual model, piloted it using cognitive interviews with people living with dementia and refined it into a shorter (37-item) version for further psychometric evaluation and data reduction [17].

**Table 1:** Domains and components from the new conceptual model of resilience in dementia (from Windle et al. 2023)

Resilience
*Individual resources*
Psychological strengths
Maintaining sense of humour
Positivity, gratitude, hope and optimism
Acceptance of the diagnosis
Focus on what you can do
Openness about diagnosis
Faith or religious beliefs
Live for the day/in the present
Comparison to others less fortunate
Practical approaches for adapting to life with dementia
Maintaining pre-diagnosis activity
Adapting to new lifestyle/changing abilities
Comfort in the ordinary (e.g., listening to music/TV/coffee)
Practical adaptation
Educating oneself/seeking information
Continuing with hobbies, interests, and activities
Participating in hobbies and activities
A sense of purpose
*Community resources*
Strong relationships with family and friends
Supportive carer
Support from family
Contact with others
Peer support and education
Advocacy and educating others about dementia
Joining and being part of a group
Support from peers (living with dementia)
Participating in community activities
Supportive community resources
Religious activity
*Societal resources*
The role of professional support services
Positive connections with healthcare professionals

### Aims

This third phase of measure development is the focus of the present study, in which we conducted preliminary field-testing and psychometric evaluation. We administered the draft 37-item resilience measure and undertook psychometric evaluation (construct validity, internal consistency, and convergent validity with other measures) in order to produce a final (shorter) resilience outcome measure for future use in research, policy and practice.

## Methods

### Ethical approval

The study received a favourable opinion from the Bangor University Healthcare Sciences Ethics and Research Committee (2023–17293) and is part of the wider RDS Impact Study, approved by the University College London Research Ethics Committee (8545/004: Rare Dementia Support Impact Study; [18]).

### Participants

We aimed to recruit 185 people living with dementia to complete the 37 resilience measure questions in the survey, using an item-to-participant ratio of 1:5 [19]. Inclusion criteria included being over 18 years old, living with a diagnosis of dementia, living in the UK, able to read and understand English, and able to provide informed consent.

Participants were recruited March-August 2023. The study was widely advertised through several dementia networks, including Rare Dementia Support, North Wales Dementia Network, the UK Network of Dementia Voices (DEEP: dementiavoices.org.uk), Centre for Ageing & Dementia Research (CADR Cymru: www.cadr.cymru) and targeted advertising to people living with dementia who are part of Join Dementia Research (joindementiaresearch.nihr.ac.uk). The study was also advertised on social media and in-person meetings, for example, support groups.

### Survey design

This is a survey study that was predominately conducted online but was also available as a paper survey if preferred (available in standard and large font sizes). In keeping with the DEEP guide on writing dementia friendly information [20], clear and concise language was used to maximise clarity and conciseness.

The participant information sheet and consent form were available in both English and Welsh for those living in Wales, in accordance with the Active Offer principle in Wales (whereby a service is offered in Welsh without someone having to ask). Due to the stage of measure development, the questionnaire was only available in English.

An online survey was created using the software Qualtrics, and equivalent paper copies were generated. The questionnaire (available in Additional File 1) comprised 10 demographic questions, the 37-item resilience measure, with scores ranging on a 5-point scale from 1 = strongly disagree to 5 = strongly agree, with an additional option of ‘not applicable’, and two scales for assessing convergent validity—The 7-item Generalised Anxiety Disorder Assessment (GAD − 7) [21], with scores ranging on a 4-point scale from 0 = not at all to 3 = nearly every day, and the 5-item Canterbury Wellbeing Scale (CWS) [22] with scores ranging from 0–100 for each item, leading to a composite wellbeing score between 0 and 500.

Three people living with dementia piloted the survey before recruitment commenced. They accessed the survey online, with or without support from a carer, and with no time restrictions. Suggestions for improvement were made via email, phone, or in person, depending on individual preference. Changes made following feedback included changing ‘proceed’ to ‘continue’ on the first page, changing the background of the questionnaire to a pale pastel colour (from white), and amending the ‘who do you live with’ question so that multiple boxes could be ticked. Moreover, the instructions for the resilience questions, which had initially appeared only at the beginning of the section, were added to the top of each page of resilience items (3 questions were displayed per page).

### Procedure

Participants first reviewed the information sheet and consented by confirming their eligibility and their consent to take part. This was made a requirement in the online survey, so participants were not able to complete the questionnaire without opting in. In accordance with the Mental Capacity Act [23] participants were assumed to have capacity. It was implied by participants’ ability to navigate the platform, acknowledge that they had read and understood the information sheet, and complete the survey that they were able to weigh up and understand the task they were being invited to participate in. Participants could refuse to participate or withdraw at any time but answers up until that point were stored and analysed unless otherwise requested.

The survey was expected to take 20–60 min to complete. Participants were informed that they could take a break if needed. The order of presentation of items for the resilience item pool were randomised both in Qualtrics and paper versions between participants to reduce the impact of survey order effects and response fatigue. As validated measures, the question order of the GAD- 7 and Canterbury Wellbeing scales were not randomised. The demographics questions were presented in typical order for a demographics section, for ease of completion. To minimise the risk of unintentional missing data, the online survey prompted participants for a response if an item was missed (this applied to all items in the survey); but if a person intentionally missed the question, they could still proceed without answering.

While the risk of harm or distress due to participation in this study was low, contact details of organisations that can provide support were provided at the end of the questionnaire (see Additional File 1), and participants were advised to contact their doctor if they had concerns about their mental or physical health.

Questionnaires could be completed anonymously. However, respondents were offered a £20 shopping voucher for their participation, and for this a postal address was requested (no personal information was retained after posting the vouchers). All electronic data were stored in encrypted folders and all physical data in a locked unit. No personal identifiers were stored with the data to ensure that data files were anonymous.

### Data analysis

#### Stage 1: data cleaning

Qualtrics data were exported into SPSS version 29 and data from paper versions added. The data were screened by JR and the following criteria applied for exclusion: a) pre-recruitment feedback entries (initial feedback entry of n = 3 who made suggestions for improving survey); b) declined to proceed (at consent level); c) no responses (clicked ‘continue’ at consent level but did not complete any survey questions); d) duplicate – respondent completed the questionnaire more than once (first or most complete response was retained); e) 90% + resilience items missing; f) ‘Straight-lining’ (answering all items the same to finish quickly); and g) fraudulent (identified using a combination of the following –- email address entry as opposed to postal for vouchers, longitude-latitude information, IP address, formatting of dates in text entries, large number of similar entries simultaneously). Decisions were verified by CM.

#### Stage 2: initial item reduction of items that are similarly worded

The conceptual model on which the initial items were based and described in Windle et al., [16] includes 7 domains of resilience with 24 components (See Table 1). The first stage of the process of measure development [17] yielded an item pool of 37 questions to be considered going forward. Components may have been represented by more than one item, with some items similarly worded (e.g., ‘There are lots of people worse off than me’ and ‘There is always someone else worse off than me’).

The data were examined to understand how participants responded to these similarly worded or ‘paired items’ with a view to reducing items to one per component category to avoid unnecessary repetition and a longer, more burdensome questionnaire. The following criteria were set for inclusion of one item from a category: 1) Keep if only item in a category of the conceptual model (i.e. no paired items); 2) Keep if full range of response options used for one item and not the other in a category (i.e. strongly disagree – strongly agree); 3) Keep if fewer ‘seen but missing’ for one item (i.e., if every respondent answered one item but not the other within a category); 4) Keep if distribution of one item is closer to normal, with skewness and kurtosis scores closer to zero.

In instances where the descriptive statistics indicated that each item from a pair differed in meaning (i.e., the response patterns differed), both items were retained. If the abovementioned criteria did not clearly identify one item for removal (e.g., both performed almost identically, or contrasting criteria were present), a sensitivity analysis was run in the next stage to determine which was the best fit.

#### Stage 3: psychometric evaluation—construct validity

Our previous work suggests that resilience is multidimensional [16, 17], and the set of items used in this work were purposefully selected to reflect the range of dimensions identified by the conceptual model. Therefore, exploratory factor analysis (EFA) using the Principal Axis factoring method with Varimax rotation was undertaken using SPSS (Version 29) to uncover the underlying factor structure behind the items [24].

Prior to performing EFA, the bivariate correlation matrix of all items was analysed to identify potential multicollinearity between pairs of items, with items removed if r > 0.8. To further identify potential multicollinearity or singularity issues the determinant of the correlation matrix was examined, with a value of > 0.00001 considered suitable for analysis [25]. Adequacy of data for EFA was tested using the Kaiser–Meyer–Olkin (KMO) [26] test, with a minimum acceptable value of 0.5. Bartlett's test of sphericity verified whether there was a relationship between variables [27]. Missing data were excluded pairwise.

Factors with eigenvalues ≥ 1 were deemed significant. Items with factor loadings below 0.4 were supressed, and items that cross-loaded (loading > 0.40 on more than one factor) were considered inadequate and removed [28–30]. Items with communalities < 0.25 were removed (higher than the cut-off of < 0.2 recommended by Child, [31]). All retained factors were to have ≥ 3 items each loading higher than 0.4, and not cross-loading (defined by an item loading > 0.4 on more than one factor) on any other factor. Once a decision was made on the final model, a label was given to each factor/domain upon agreement among all authors. The internal consistency of the scale and subscales derived from the factor analysis was then ascertained using Cronbach’s Alpha with pairwise deletion (as per EFA), performed in R [32]. An alpha coefficient of 0.60 or 0.70 is deemed an acceptable threshold for reliability, but 0.80 is preferred for the psychometric quality of scales [28, 33].

#### Stage 4: scoring methods and convergent validity

Eight scoring methods were explored including different potential methods for dealing with not applicable (N/A) or missing responses. We aimed to identify the best method that would be least onerous on anyone using the scale in practice in the future. First, we calculated the sum of all Likert responses. This approach is commonly used in other measures of resilience, such as the Resilience Scale (RS- 14) [34], the Connor-Davidson Resilience Scale (CD-RISC) [35] and the Adult Resilience Measure (ARM) [36]. This approach may be the most straightforward but does not deal with missing values, thus potentially producing a biased lower score if any missing data exists. Second, we performed a neutral-value substitution, in which missing data were assigned a neutral Likert score of 3 to reflect a ‘neither agree or disagree’ response. Third, we explored imputing a person’s overall mean score for the scale to missing data [37]. Whilst this method may be complex to use in practice, we sought to compare its outcome with the other, more straightforward, approaches. Fourth, we performed a complete case analysis, where cases that included missing data were omitted.

We then calculated the sum of the mean responses for each domain (method 5). This method calculated the mean for each domain by the number of completed responses and is the approach used in the Resilience Scale for Adults (RSA) [38]. As above, we then performed sum of means analyses using neutral-value substitution (method 6) and person’s mean substitution (7) for missing data, followed by complete case analysis (8).

In assessing convergent validity, a negative correlation was hypothesized between resilience and GAD- 7 and a positive correlation between resilience and well-being (CWS). These are based on findings from people living with dementia for similar constructs. For example self-efficacy, optimism and self-esteem were positively associated with quality of life, well-being and life satisfaction [9], and self-efficacy and quality of life were negatively associated with anxiety [39]. Based on the COSMIN recommendations for correlations with instruments measuring related, but dissimilar constructs [40] we expected these to be between 0.30‐0.50.

## Results

### Stage 1: data cleaning

**Fig. 1:**
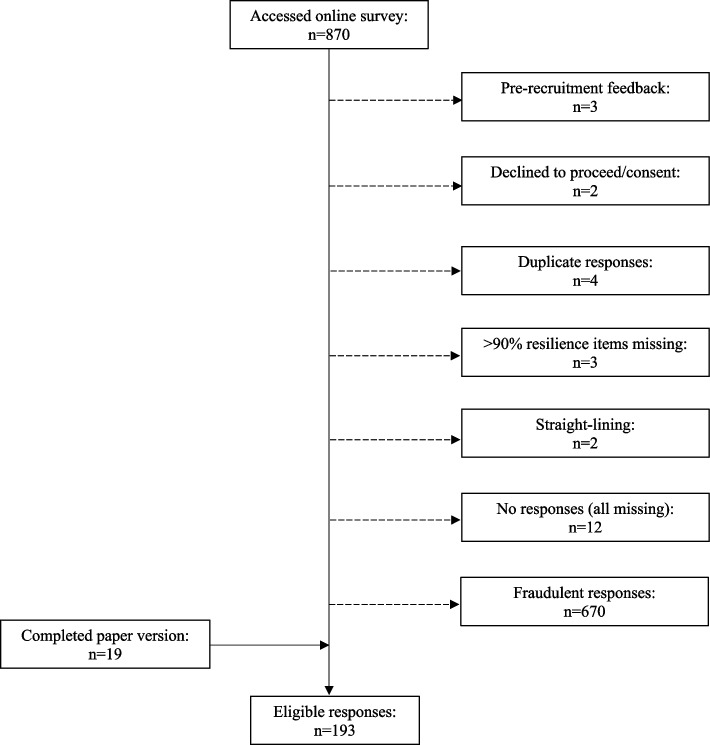
Flow through study access

Figure 1 depicts the flow through study access. In total, 193 eligible responses were recorded (n = 174 online; n = 19 on paper).

Table 2 demonstrates the demographic characteristics of participants. A range of diagnoses were present in the sample, including *n* = 131 people living with more typical forms of dementia (Alzheimer’s Disease, Vascular Dementia, or a combination of the two), *n* = 5 where no specific diagnosis was given, and *n* = 57 with rarer forms of dementia (all other diagnoses represented in Table 2). The sample was 58% male and 99.5% Caucasian.

**Table 2:** Demographic information about participants

		N
Sex		
	Male	113
	Female	80
Age		
	Mean	69.9
	SD	9.5
	Range	47–93
Marital status		
	Single	10
	Married	149
	Cohabiting	9
	Widowed	11
	Divorced/separated	14
Living arrangements		
	Lives alone	27
	With spouse/partner	156
	With child(ren)	18
	With parents	1
	With other family members	8
	With others in assisted living accommodation	2
	Other	2
First language		
	English	182
	Welsh	8
	Other	3
Ethnic group		
	White	191
	Mixed or multiple ethnic groups	1
	Missing data	1
Education		
	Primary school	2
	Secondary school	57
	Higher education	101
	Training or apprenticeship	32
Current occupation		
	Employed or self employed	12
	Retired	150
	Looking after home/family	6
	Long term sick or disabled	22
	Other	3
Dementia diagnosis		
	No specific diagnosis was given	5
	Alzheimer’s Disease (AD)	82
	Vascular dementia (VD)	20
	Mixed (Alzheimer’s disease & vascular)	29
	Lewy body dementia (LBD)	11
	familial Alzheimer’s disease (fAD)	1
	familial Frontotemporal dementia (fFTD)	2
	Frontotemporal dementia (FTD)	10
	Primary progressive aphasia (PPA)	6
	Logopenic aphasia (LPA)	3
	Progressive nonfluent aphasia (PNFA)	1
	Semantic dementia (SD)	2
	Posterior cortical atrophy (PCA)	11
	Other rare dementias (specified by participants)	10

### Stage 2: initial item reduction of items that are similarly worded

The initial item removal exercise resulted in decisions to remove 13 items from pairs representing component categories; to keep 18 single items (both from pairs and items where there was one question representing a component); and to keep both items from 3 pairs (n = 6) where descriptive statistics indicated that the items may differ in meaning to each other. This led to a reduced 24-item draft resilience measure. The decision process is available in Additional File 2.

### Stage 3: psychometric evaluation—Construct validity

EFA using the principal axis factoring method and Varimax rotation was conducted on the refined item pool of n = 24 resulting from stage 2, to establish the structure of the new resilience measure. Moreover, sensitivity analyses were performed on 3 pairs where the criteria did not clearly identify one item for removal in Stage 2: pair 3 (Q17 ‘I have accepted my diagnosis’; Q18 ‘I accept my condition’), pair 4 (Q20 ‘There are still lots of things I can do’; Q21 ‘I do the best I can’), and pair 21 (Q49 ‘The support I receive from health and social care professionals meets my needs’; Q50 ‘I am happy with the support I receive from health and social care professionals’). This was achieved by swapping each item in and out of the EFA data set, and all with combinations.

No multicollinearity issues were identified from the bivariate correlation matrix with all items correlating at r < 0.8, nor from the determinant of the correlation matrix (= 0.002). Bartlett’s test of sphericity confirmed correlations between items were sufficiently large (χ2 [171] = 958.47, *p* < 0.001). The Kaiser–Meyer–Olkin measure of sampling adequacy was 0.803 (a value deemed as ‘great’ according to Sofroniou & Hutcheson [41]), confirming that EFA was appropriate with this data.

EFA via principal axis factoring and varimax rotation resulted in the elimination of 5 items. Item Q49 ‘The support I receive from health and social care professionals meets my needs’ did not load above 0.4 on any of the factors. Item Q43 ‘My social life is satisfying’ often cross-loaded between two factors, or loaded onto factors with ≤ 3 items, and a decision was made to remove it. Item Q48 ‘My personal beliefs help me live with my dementia (For example: faith, religion, spiritual beliefs)?’ had a communality of < 0.25 and was removed. Item Q33 ‘I find information that helps me live with dementia’ performed inconsistently, loading on to different factors during the EFA and a decision was made to remove it. Item Q44 ‘Educating other people about my dementia is important’ did not load above 0.4 on any factor including 3 or more items in any analyses.

Sensitivity analyses suggested ‘I accept my condition’ (Q18) to be more suitable than ‘I have accepted my diagnosis’ (Q17); ‘There are still lots of things I can do’ (Q20) as more suitable than ‘I do the best I can’ (Q21); and both items relating to support from health and social care professionals (Q49 and Q50) were consistently removed in all analyses due to not loading above 0.4 on any factors.

After removing items loading < 0.4, unstable items, and items with low (< 0.25) communality, a 5-factor solution stabilised, explaining 46.17% of the variance. In terms of reliability, the Cronbach’s alpha of the whole scale was 0.85 (with a confidence interval of 0.81–0.88), which is considered ‘good’ [28]). The five factors resulting from the EFA were named: ‘outlook’ (α = 0.75), ‘adaptation’ (α = 0.78), ‘acceptance’ (α = 0.69), ‘community and peer support’ (α = 0.78) and ‘family and friends’ (α = 0.62). Table 3 represents the EFA and structure of the final measure.

**Table 3:** The EFA and structure of the final measure

		Individual			Community	
		**Outlook**	**Adaptation**	**Acceptance**	**Community & Peer support**	**Family & friends**
1	Taking a positive attitude helps me manage	0.688				
2	I can see the funny side of things	0.613				
3	There are lots of people worse off than me	0.519				
4	I take each day as it comes	0.476				
5	I find enjoyment in everyday things	0.434				
6	I keep up my hobbies and interests		0.706			
7	I do things important to me		0.633			
8	I find ways around problems in my life		0.625			
9	There are still lots of things I can do		0.531			
10	Learning about dementia has helped me to live with it			0.565		
11	I am open with other people about my dementia			0.536		
12	I accept my condition			0.522		
13	Making changes helps me live with my dementia			0.444		
14	I get support from other people experiencing similar challenges				0.775	
15	I am part of a supportive community (For example: online or face to face groups, forums, clubs and societies)				0.680	
16	Meeting other people going through difficulty has shown me I am not on my own				0.670	
17	My family is supportive					0.746
18	I am happy with the support I receive from my partner					0.633
19	I feel supported by my friends					0.468

Extraction Method: Principal Axis Factoring. Rotation Method: Varimax with Kaiser Normalization

^a^Rotation converged in 7 iterations

### Stage 4: scoring methods and convergent validity

Eight methods of scoring were carried out on the new 19-item measure.

Calculating the sum score of the measure yielded a potential score between 19 and 95. Using this method without addressing missing values gave a mean score of M = 73.17 (SD = 9.63, range 38–95). Although perhaps the most straightforward approach this will lead to a reduced score if any item has not been answered. Calculating the sum score using imputation, either through substitution of missing values with a neutral value (i.e., a score of 3; M = 74.24, SD = 8.98, range = 44–95) or with the person’s overall mean score (M = 74.54, SD = 9.17, range = 43.28–95), slightly increases the overall sum score.

Calculating the sum of the means for each of the 5 domains/factors produced a potential score between 5 and 25. Following this approach without using imputation yielded a mean score of 19.42 (SD = 2.45, range = 11.32–25). Using neutral-value substitution (M = 19.38, SD = 2.38, range = 11.48–25) and the person’s mean value (M = 19.48, SD = 2.432, range = 11.24–25) produced similar outcomes.

Complete case analyses, whereby only data from respondents who answered all questions, reduced the sample size considerably (*n* = 148). The trend was the same as the other methods of analyses, but with a loss of power due to reduced sample size.

To add rigor to the findings, EFA was repeated on the final measure using scoring methods that involve imputation (neutral-value and person’s mean). An identical factor structure was provided by both.

Table 4 provides resilience scores, and correlation and significance values for the CWS and GAD- 7 using different potential methods of scoring the resilience scale. Complete valid responses were provided by *n* = 189 participants on the Canterbury Wellbeing scale (M = 326.73, SD = 99.26), and n = 193 on the GAD- 7 (M = 6.233, SD = 5.71). Assessment of convergent validity yielded a positive correlation between resilience and well-being (CWS). A negative correlation was observed between resilience and anxiety (GAD- 7). These were as expected, between 0.30‐0.50 (apart from complete case analysis), based on the COSMIN recommendations for correlations with instruments measuring related, but dissimilar constructs [40]. All correlations were significant, apart from that between the GAD- 7 and complete case analysis of the sum of means for each domain. For resilience and well-being these ranged from 0.44 to 0.52, and for anxiety from − 0.16 to − 0.3, depending on the scoring method utilised.

**Table 4:** Convergent validity analyses using different approaches to scoring the resilience measure

		Resilience scores					GAD- 7 (n = 193)		CWS (n = 189)	
		N (valid)	Mean	SD	Min	Max	Corr	Sig	Corr	Sig
*Sum score analyses*										
Sum score		193	73.17	9.626	38	95	− 0.3	< 0.001	0.51	< 0.001
With neutral-value substitution		193	74.24	8.982	44	95	− 0.3	< 0.001	0.52	< 0.001
With person's mean substitution		193	74.54	9.166	43.28	95	− 0.3	< 0.001	0.51	< 0.001
Complete case analysis		148	74.68	8.623	51	95	− 0.19	0.02	0.469	< 0.001
*Sum of means analyses*										
Sum of means of each domain		193	19.42	2.453	11.32	25	− 0.277	< 0.001	0.49	< 0.001
With neutral-value substitution		193	19.38	2.374	11.48	25	− 0.27	< 0.001	0.49	< 0.001
With person's mean substitution		193	19.48	2.432	11.24	25	− 0.27	< 0.001	0.49	< 0.001
Complete case analysis		148	19.52	2.276	13.32	25	− 0.16	0.06	0.436	< 0.001

## Discussion

This study reports on the preliminary field testing and validation of the first resilience outcome measure designed specifically for people living with dementia. Following gold standard procedures promoted by Streiner et al. [15] and quality standards for study design by COSMIN [40] ensured rigour of the process. This included establishing the need for a new resilience outcome measure [14], developing a conceptual model of resilience [16] and the development of an initial item pool of questions for the resilience measure [17] which involved people with dementia throughout the process. Building on these preceding phases, the work described here has led to the original 19-item ‘Bangor Dementia Resilience Scale’.

It is difficult to draw comparisons between the Bangor Dementia Resilience Scale and other resilience outcome measures, as this is the only measure that reflects the different domains of resilience as revealed in the theoretical development [16]. The 5-factor model resulting from the EFA named ‘Outlook’, ‘Adaptation’, ‘Acceptance’, ‘community and peer support’ and ‘family and friends’ correspond to the 7 domains described in the conceptual model of resilience in people living with dementia, where ‘psychological strengths’, ‘practical approaches for adapting to life with dementia’, ‘continuing with hobbies, interests and activities’, ‘strong relationships with family and friends’, ‘peer support and education’, and ‘participating in community activities’ were important for resilience [16]. The internal consistency assessment suggests that all 19 items of the Bangor Dementia Resilience Scale measured the same construct, as indicated by a good Cronbach’s alpha of 0.85.

All scoring methods and convergent validity analyses yielded the same pattern of results. Complete case analysis demonstrated the importance of handling missing data rather than excluding cases with missing data, which may introduce bias and a resultant different underlying factor structure. Due to its consistency with other resilience measures [38] and relative user-friendliness, we recommend using the sum of means of each domain (without imputation), calculating the mean for each domain by the number of completed responses and adding these means together for a score out of a potential 25. Further work is needed to establish whether domains could be used as separate subscales. We therefore recommend using the scale as a whole.

The items corresponding to the theoretical domain ‘the role of professional support services’ in Windle et al. [16] did not load above 0.4 on any factor in the EFA, and therefore the scale lacks this detail. Professional support services and interventions are important for fostering resilience in people living with dementia [42]. In the current measure it may have been that items were not worded adequately, or may have been too broad, with participants experiences differing across services and providers. Future work may wish to explore the societal level of resilience for people with dementia, in terms of support from services and organisations, in more depth.

Strengths and limitations are present within the sample of the study. The involvement and guidance from people living with dementia throughout all stages of the development of the new measure was vital. In the present study, as well as 193 people living with dementia giving their time and expertise to complete the questionnaire, people living with dementia also provided guidance around adaptations to the survey to make it more accessible, prior to recruitment. Adding to the originality of this work is that people with rarer dementias were involved throughout, ensuring the measure is more inclusive and representative.

One of the limitations of our sample is the lack of diversity in relation to people’s ethnic background, with 99.5% of participants reporting having Caucasian ethnicity. Whilst lack of diversity is common in dementia research [43], it is important to capture the experiences of people from different backgrounds and cultures and it is particularly important that communities are involved in adaptations and translations of measures into other languages [17]. The new measure would benefit from further work exploring cross-cultural adaptation and validation.

The mean resilience scores are relatively high, regardless of the scoring method. We suggest that this may be due to an inherent issue with the population who agree to take part in research studies. Lönnqvist et al. [44] suggest that research volunteers might be ‘better adjusted than nonvolunteers’ (pp.1028) and ‘healthier than the general population’ (p. 1027). If the measure is used within a healthcare setting, the mean scores may differ.

While there are various recommendations for sample size, up to a ratio of 1:20 items to participants [45], Bujang et al. [19] recognise that recruitment can be difficult in clinical settings due to prevalence of people with specific health conditions. They proposed a minimum sample of 1:3 for exploratory factor analysis, and using a ratio of 1:5 found all factor solutions in an EFA were correct for all measurement scale types. Using a 1:5 ratio, we aimed for a minimum of 185 people living with dementia to complete the 37 resilience measure questions in the survey, and exceeded this with 193 eligible responses. A total of 24 resilience items were analysed at the EFA stage, which equates to a ratio of 1:8.

The questionnaires used in this study were mostly completed online, but with the option of completing a paper copy. Online surveys may result in bias due to the non-representative nature of the population of those using the internet [46]. However, the large number of responses received suggests an increase in the number of people with access to the internet, including people living with dementia. Furthermore, the option of completing paper versions was used by some people with dementia (*n* = 19), ensuring greater access to the study. The questionnaire was self-completed at home, as opposed to during an interview, for several reasons. First, the new measure is intended to be sufficiently user-friendly to be self-complete. The second reason was that the anonymity of self-completing could mitigate social desirability response bias, as participants may respond with more honesty than in an interview with a researcher.

Participation was incentivised with a shopping voucher which is important in acknowledging and thanking experts by experience for their time and expertise [47]. However, this approach combined with advertising on social media led to many fraudulent responses (*n* = 670). We were able to identify fraudulent responses in this instance but wish to highlight the issue, as this is becoming more commonplace in online incentivised participation [48, 49]. The methods we used to combat the fraudulent responses (e.g., observing the approach to requesting vouchers, longitude-latitude information, formatting of dates in text entries, large number of similar entries arriving simultaneously) may serve as useful to other researchers undertaking research online.

Strengths and limitations also exist in the design of the study. The research survey was designed with the intention of avoiding unnecessary mental distress, with questions carefully worded and validation measures carefully selected to avoid triggering language. The order of the presentation of each questionnaire was selected to ensure participation ended on the most positively framed validation measure (the Canterbury Wellbeing Scale). However, there was a small risk that some participants may have been sensitive to the content of some questions. Participants were made aware that they were in control and could refuse to answer any question without providing a reason. They were also informed that they could take a break or stop participating completely if they wished.

Convergent validity findings were as hypothesised with a positive correlation between resilience and wellbeing, and a negative correlation between resilience and anxiety observed. These hypotheses were based on research of similar constructs from people living with dementia. Future research should explore the relationship between the Bangor Dementia Resilience Scale and various other characteristics and theoretical concepts to broaden understanding around the opportunities and limitations of resilience when living with dementia. Future research should also examine the recommended factors by undertaking confirmatory factor analysis and establish other psychometric properties, such as test–retest reliability.

### Implications for policy and practice

The measure is proposed as a self-report outcome measure. This may be completed by the person themselves or with someone supporting them, meaning it will be appropriate for people living with mild to moderate dementia. Utilisation of this new measure would enable those supporting people living with dementia to assess their resilience reliably and accurately. It has potential to be used as a tool and a conversation aid in practice, during for example, ‘What Matters’ conversations with care providers [50]. ‘What Matters’ is a conversation that establishes a person’s “current well-being, what can be done to support them and what can be done to promote their well-being and resilience for the better” [50, pp.2]. The measure has the potential to identify areas in which a person may benefit from additional support, and for planning effective strengths-based, person-centred, and family-centred care. Further, this measure could be suitable for evaluating programmes and interventions designed to improve resilience in people living with dementia, however further research is required to ascertain the extent to which the measure is sensitive to change due to service interventions.

## Conclusion

This significant work responds to an absence of resilience measures designed specifically for and with people with dementia, presenting successful preliminary field-testing and validation of the Bangor Dementia Resilience Scale. Robust methodology ensured rigor of the process and a resulting outcome measure of gold-standard quality. The Bangor Dementia Resilience Scale provides a new strengths-based measure that may be a valuable tool towards provision of good person-centred support for people living with dementia, as well as for assessing positive responses to services and interventions. The measure is available to download and use free of charge at dsdc.bangor.ac.uk/dementia-resilience-scale.

## Data Availability

The data that support the findings of this study are available from the corresponding author [JRR] upon reasonable request.
